# Supplementary material for: Ependymal cell lineage reprogramming as a potential therapeutic intervention for hydrocephalus
Source: EMBO Mol Med. 2024 Oct 28;16(11):2725–48. doi: 10.1038/s44321-024-00156-5 (PMC11555118; doi:10.1038/s44321-024-00156-5)
Supplement: Supplementary file 1 — Appendix [file 44321_2024_156_MOESM1_ESM.pdf]

Appendix

Table of Content:

Appendix Figure Sl p2

Appendix Figure S2 p3

Appendix Figure S3 p4

Appendix Figure S1

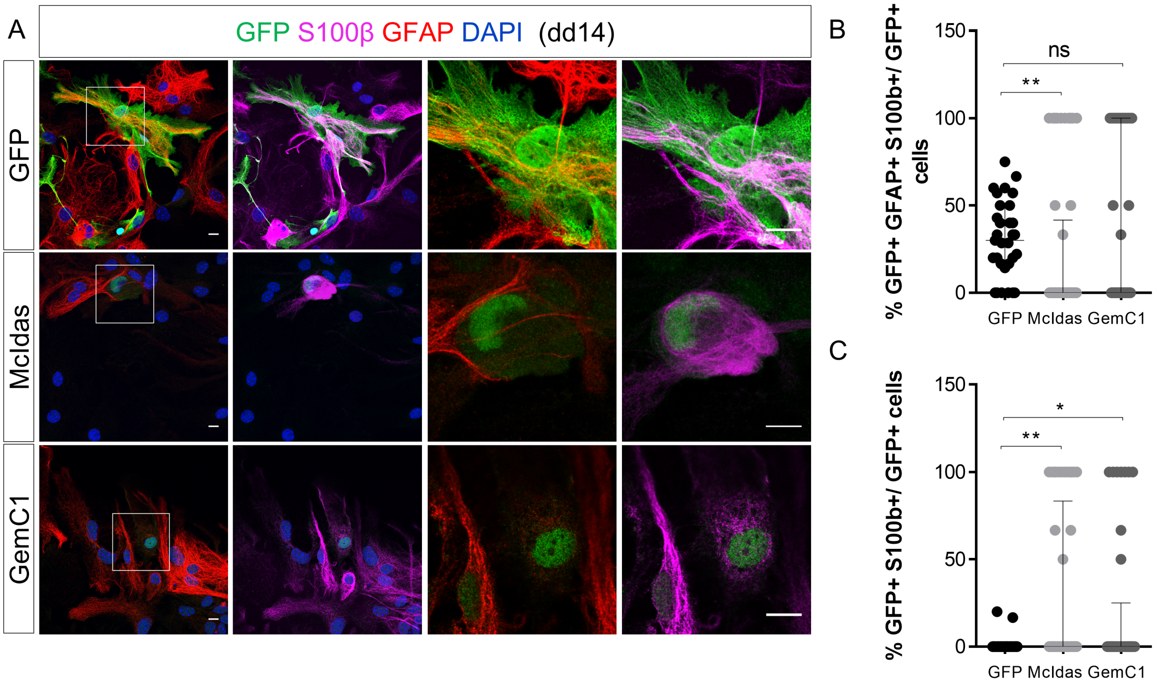

McIdas downregulates the astrocytic identity of the reprogrammed cells.

(A) Transduced cortical astrocytes, at day 14 of differentiation were immunostained using antibodies against GFP (green), to mark infected cells, S100β (magenta) and GFAP (red). Higher magnification of the boxed area is shown in the right panel.

(B-C) The graph depicts the percentage of the infected cells (GFP+) that co-express the astrocytic markers S100β and GFAP over the total number of the infected cells, which correspond to astrocytes (B). The percentage of the infected cells (GFP+) which display S100β staining around their cell body, which corresponds to ependymal cells, is depicted in the graph (C) Data are presented as the median ±interquartile range (IQR) of two independent experiments.

DNA was stained with Dapi (blue). Scale bars, 10 μm. *p<0.05, **p < 0.01

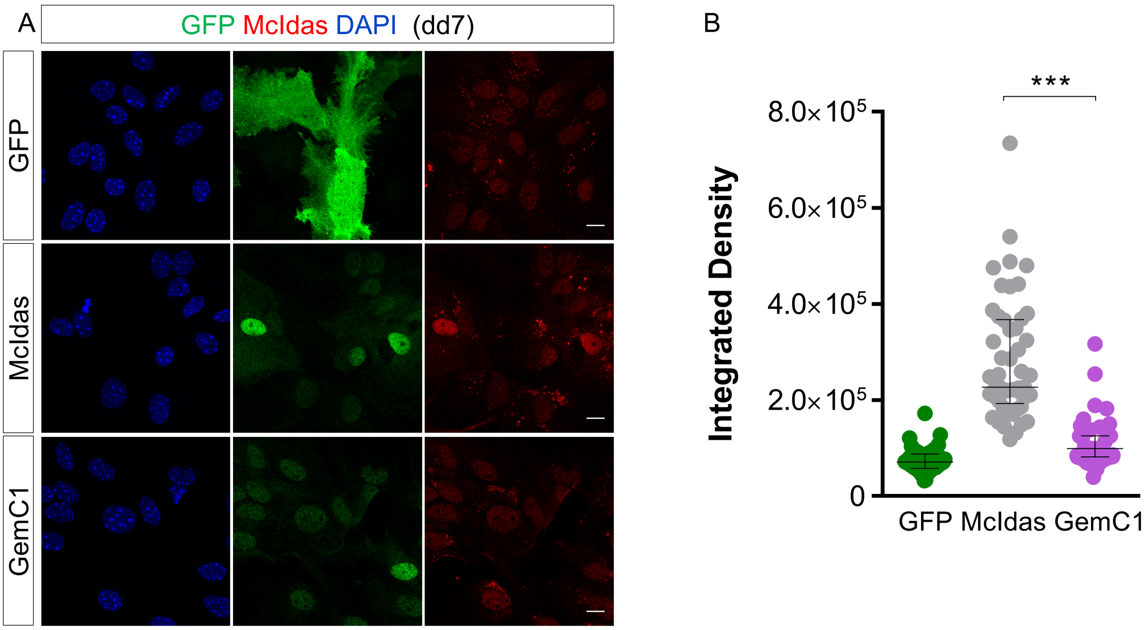

Ectopic expression of GemC1 induces low McIdas expression levels during ependymal reprogramming.

(A) Cortical astrocytes were transduced with lentiviruses encoding GFP, GFP-McIdas (McIdas) or GFP-GemC1 (GemC1). Immunofluorescence experiments were performed at day 7 of differentiation with specific antibodies against GFP (green) to mark the infected cells, and McIdas (red).

(B) The graph shows the intensity of McIdas per cell. Data are presented as the median ±interquartile range (IQR) of two independent experiments.

DNA was stained with Dapi (blue). Scale bars, 10 µm. *** $p < 0.001$.

Appendix Figure S3

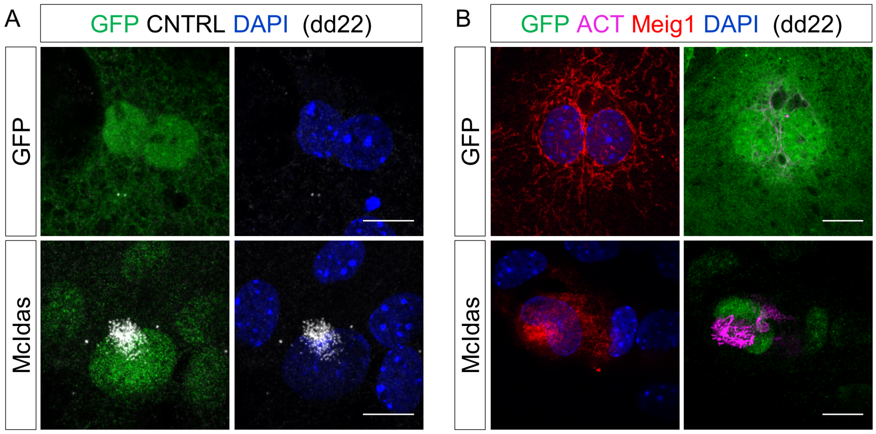

McIdas induces the ependymal characteristics in reprogrammed cortical astrocytes.

(A) Cortical astrocytes transduced with GFP or GFP-McIdas (McIdas) lentiviruses were immunostained at differentiation day 22 against GFP (green) and Centriolin (grey). Note the presence of multiple basal bodies, based on Centriolin signal accumulation, in McIdas-infected cells.

(B) Infected astrocytes were subjected to immunofluorescence using specific antibodies against GFP, acetylated-a-tubulin (ACT, magenta) and Meig1 (red). Note the accumulation of Meig1 signal, revealing the presence of multiple basal bodies in McIdas infected cells which possess multiple cilia, based on ACT signal accumulation.

DNA was stained with Dapi (blue). Scale bars, 10 µm.
