# Supplementary figures and images for: Ependymal cell lineage reprogramming as a potential therapeutic intervention for hydrocephalus
Source: EMBO Mol Med. 2024 Oct 28;16(11):2725–48. doi: 10.1038/s44321-024-00156-5 (PMC11555118; doi:10.1038/s44321-024-00156-5)

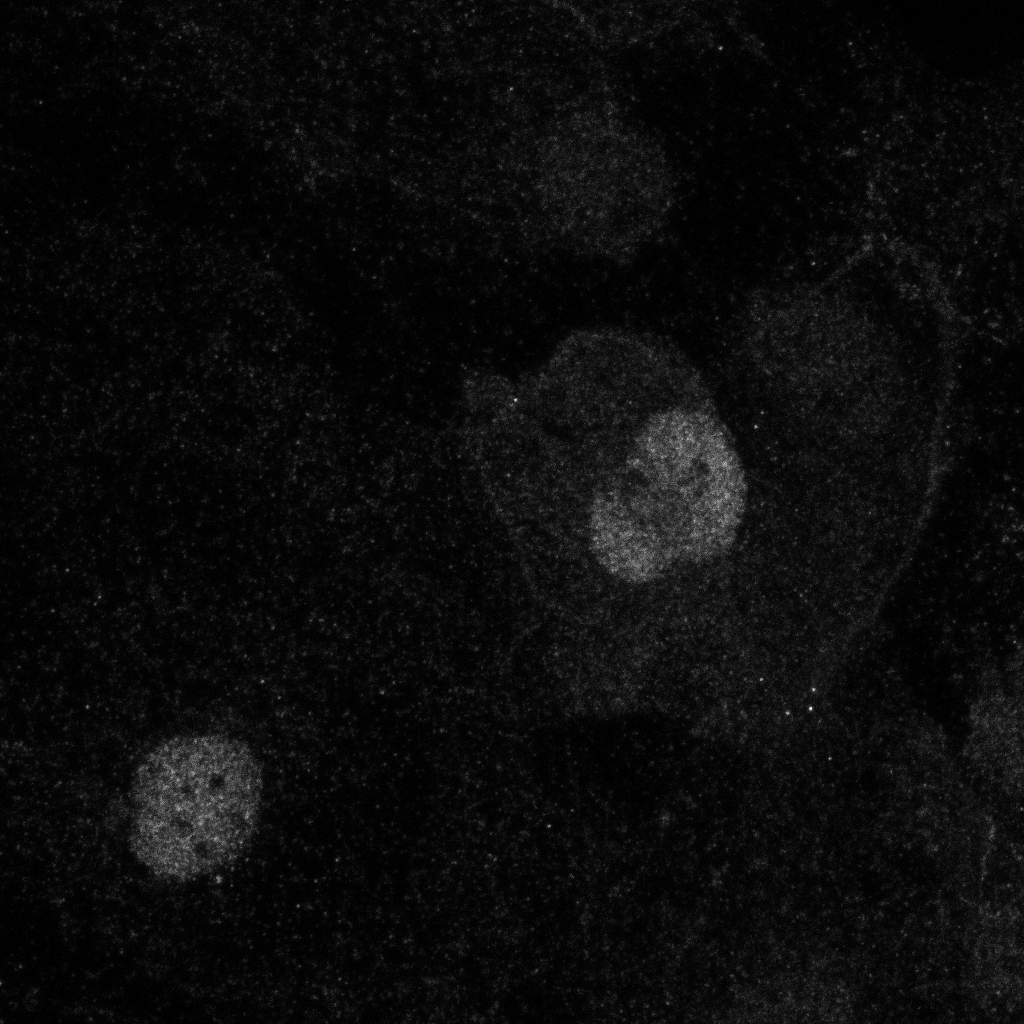

Supplement: Supplementary file 9 — Source data Fig. 1 [file 44321_2024_156_MOESM9_ESM.zip › Figure 1/Image Data 1E/1E McIdas overexpression.tif]

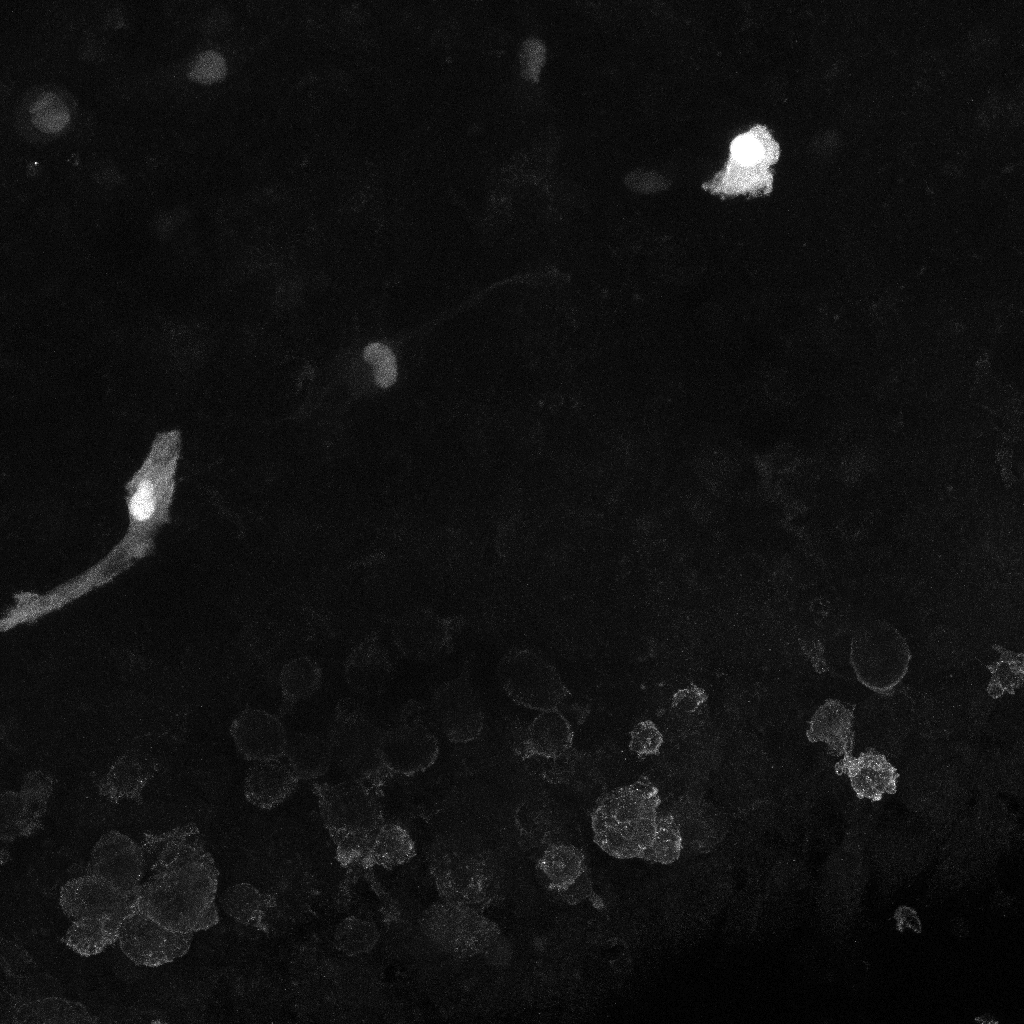

Supplement: Supplementary file 11 — Source data Fig. 3 [file 44321_2024_156_MOESM11_ESM.zip › Figure 3/Image Data 3A/3A GFP overexpression.tif]

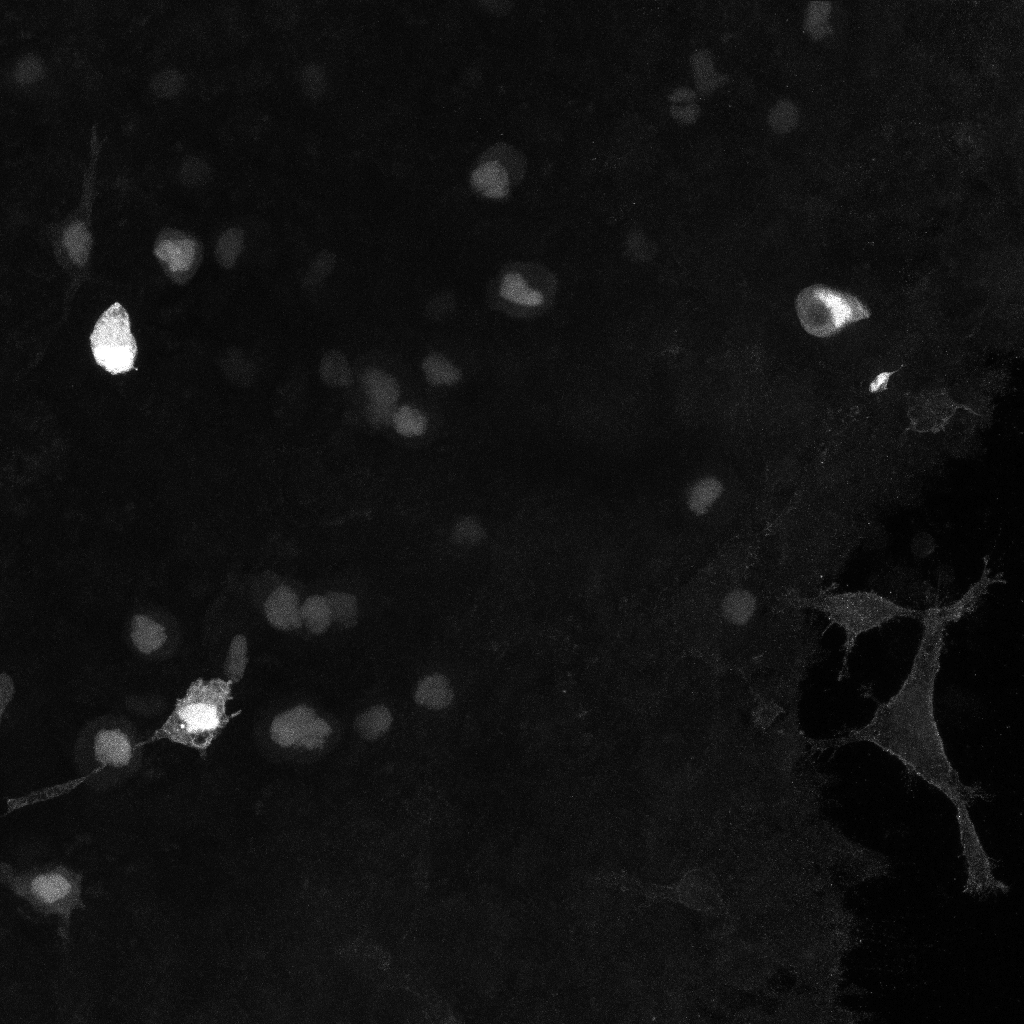

Supplement: Supplementary file 11 — Source data Fig. 3 [file 44321_2024_156_MOESM11_ESM.zip › Figure 3/Image Data 3A/3A McIdas overexpression.tif]

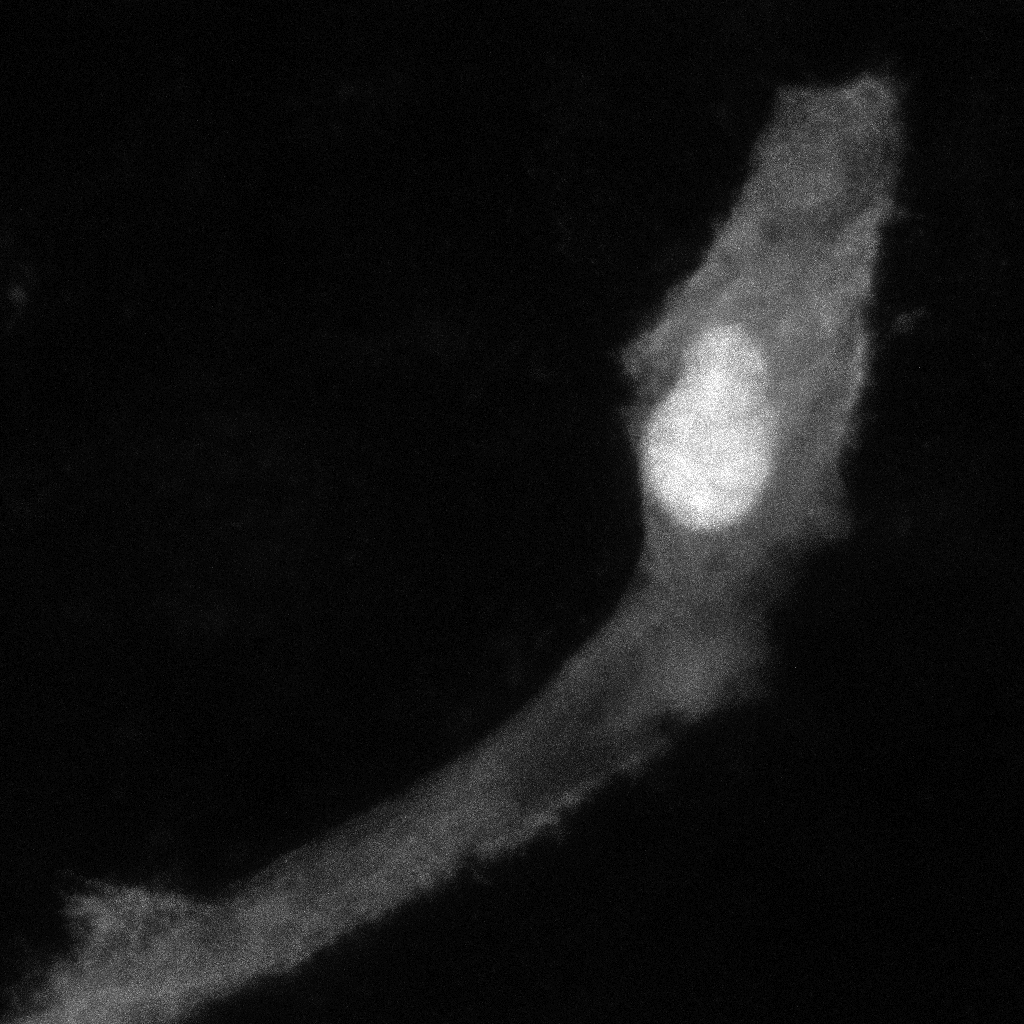

Supplement: Supplementary file 11 — Source data Fig. 3 [file 44321_2024_156_MOESM11_ESM.zip › Figure 3/Image Data 3A/3A zoom GFP overexpression.tif]

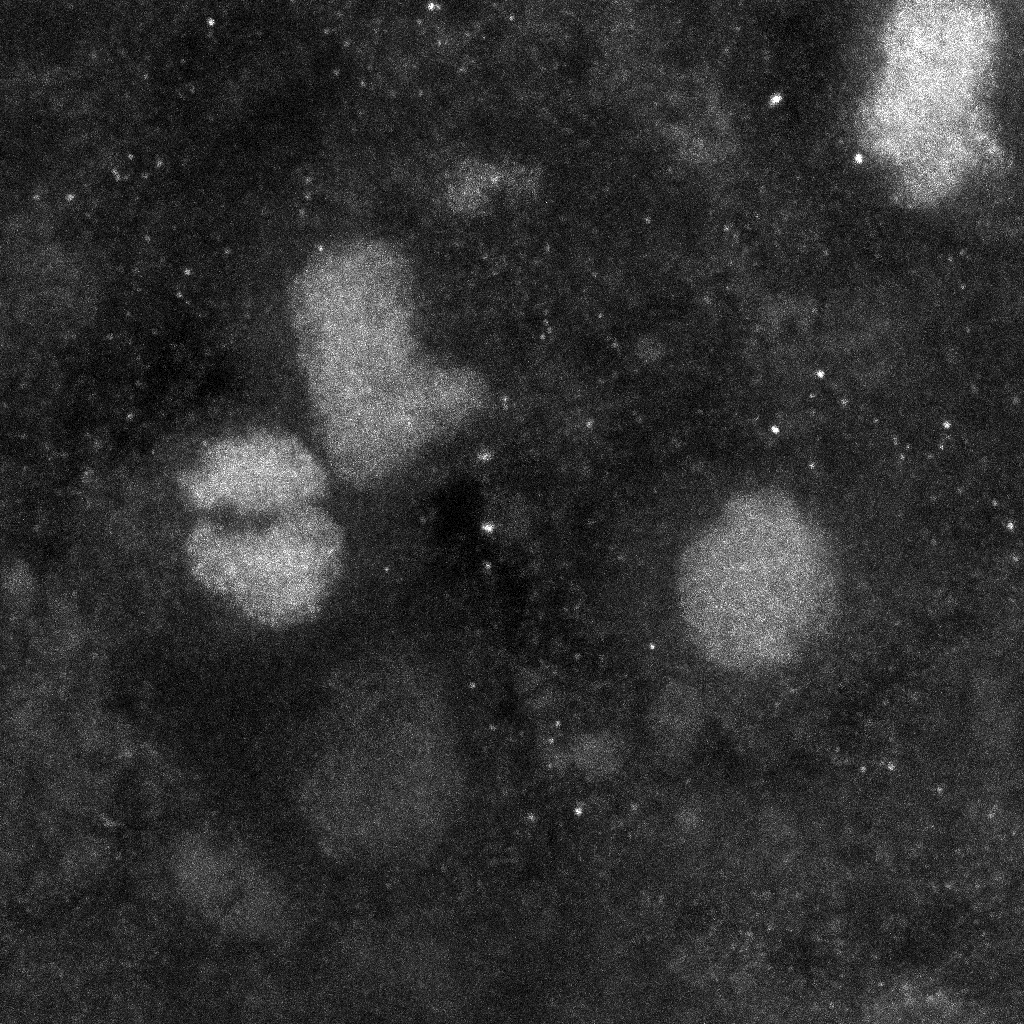

Supplement: Supplementary file 11 — Source data Fig. 3 [file 44321_2024_156_MOESM11_ESM.zip › Figure 3/Image Data 3A/3A zoom McIdas overexpression.tif]

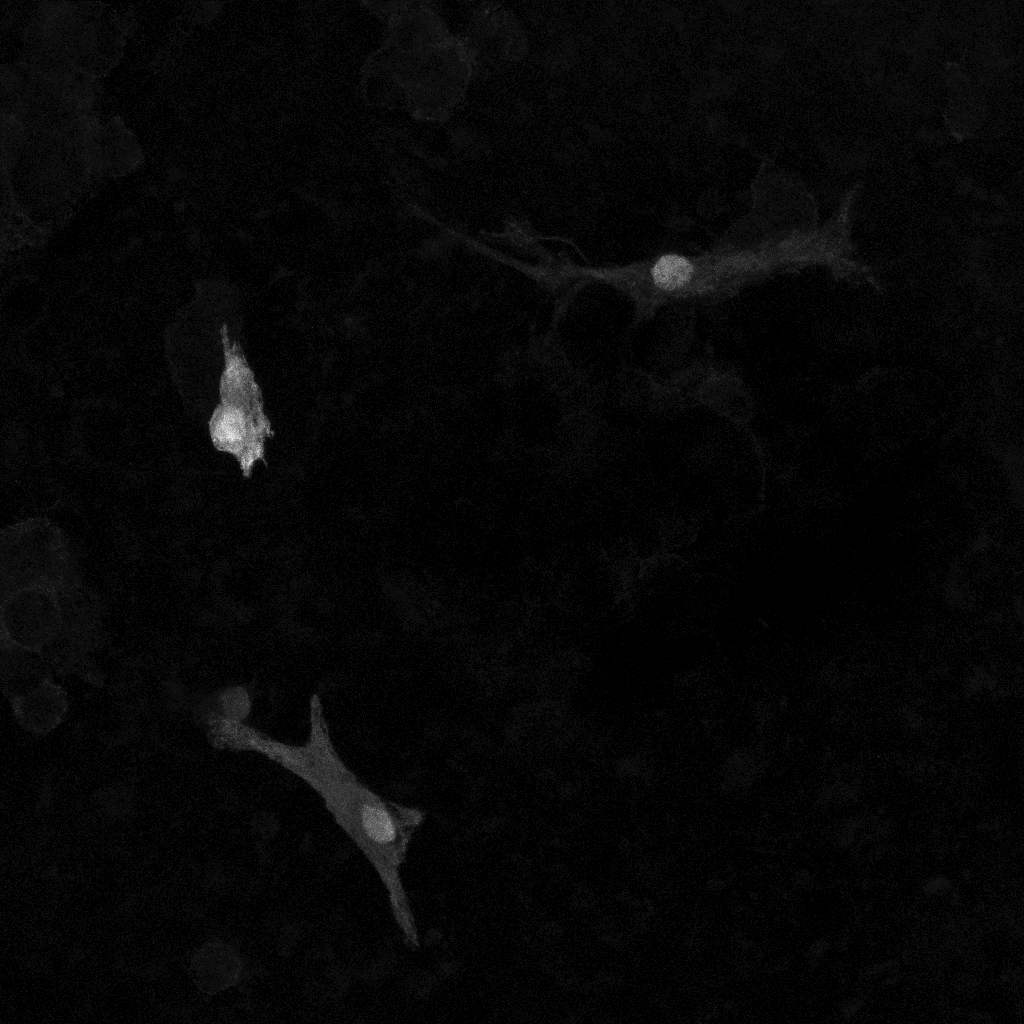

Supplement: Supplementary file 11 — Source data Fig. 3 [file 44321_2024_156_MOESM11_ESM.zip › Figure 3/Image Data 3C/3C GFP overexpression.tif]

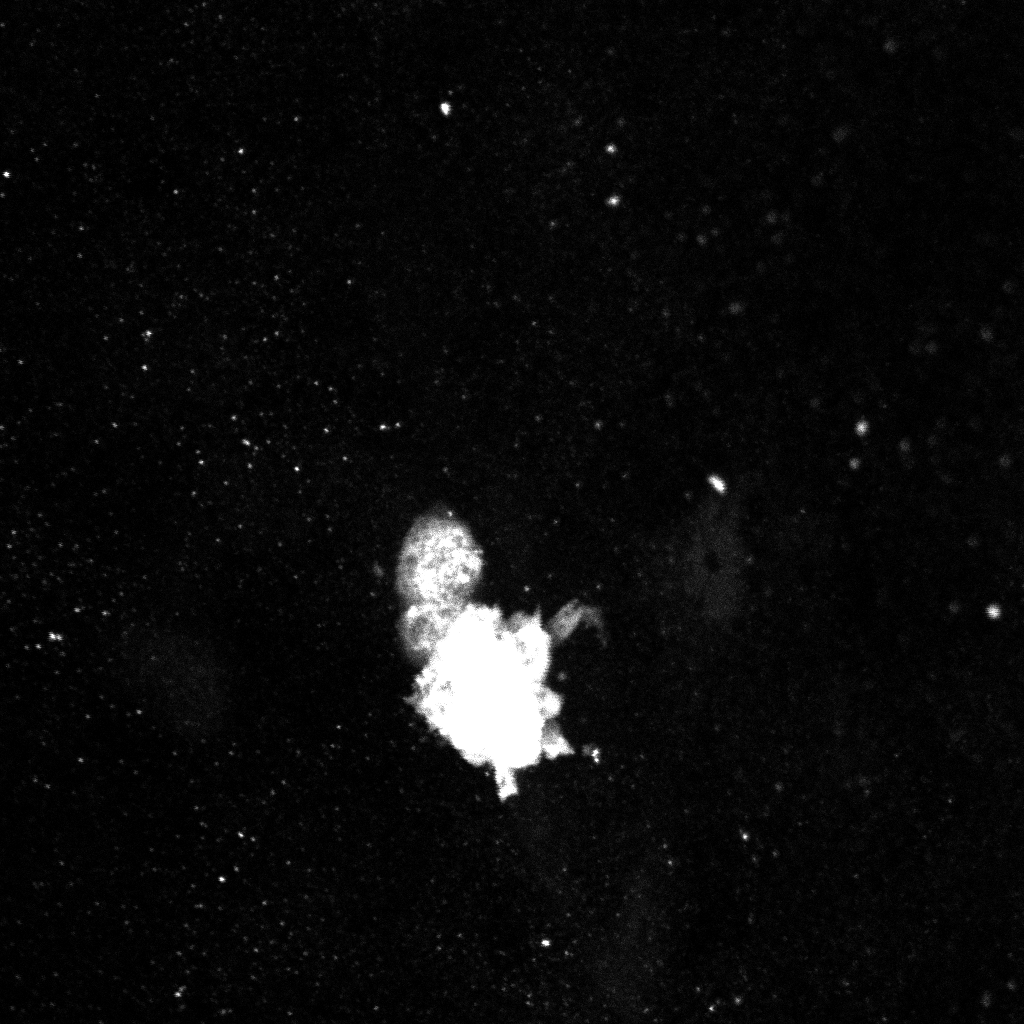

Supplement: Supplementary file 13 — Source data Fig. 6 [file 44321_2024_156_MOESM13_ESM.zip › Figure 6/Image Data 6A/6A zoom GFP overexpression.tif]

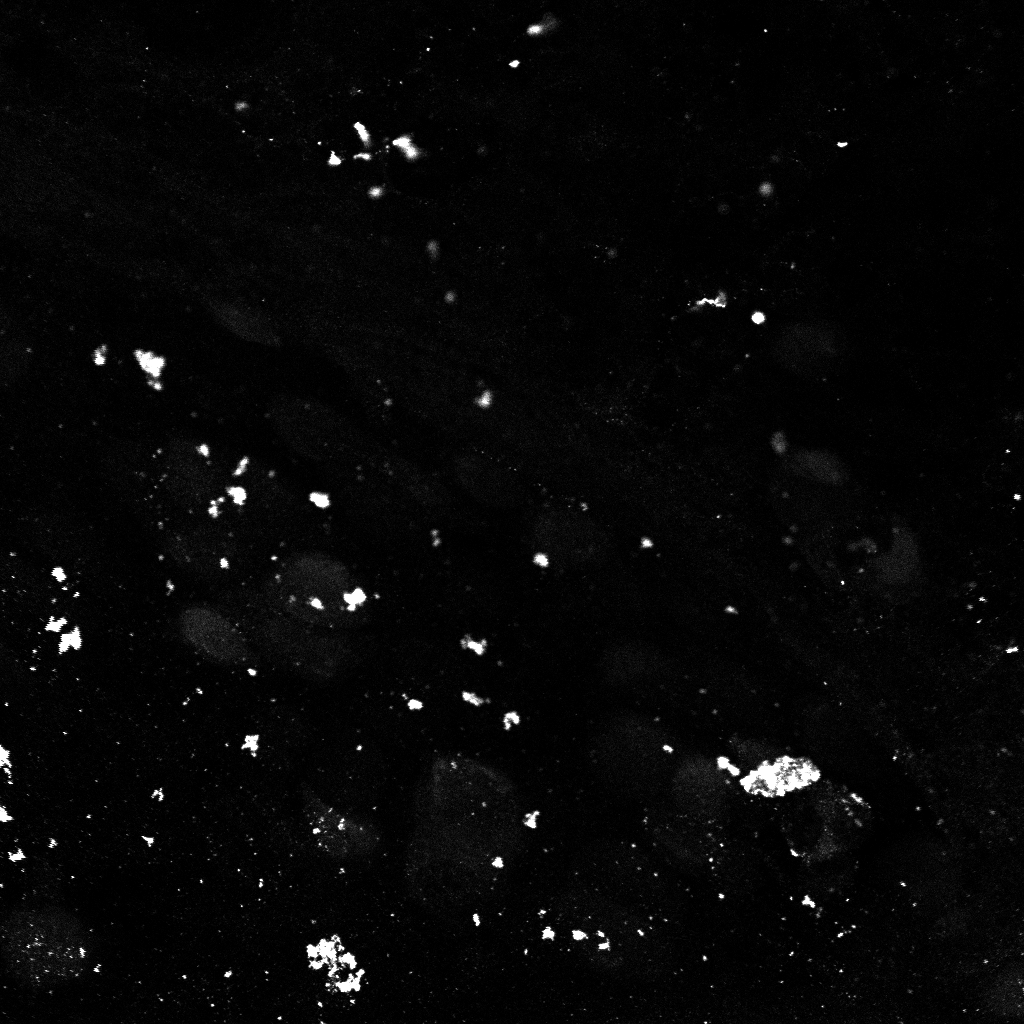

Supplement: Supplementary file 13 — Source data Fig. 6 [file 44321_2024_156_MOESM13_ESM.zip › Figure 6/Image Data 6B/6B McIdas overexpression.tif]
