# Supplementary material for: Ependymal cell lineage reprogramming as a potential therapeutic intervention for hydrocephalus
Source: EMBO Mol Med. 2024 Oct 28;16(11):2725–48. doi: 10.1038/s44321-024-00156-5 (PMC11555118; doi:10.1038/s44321-024-00156-5)
Supplement: Supplementary file 15 — Expanded View Figures [file 44321_2024_156_MOESM15_ESM.pdf]

Expanded View Figures

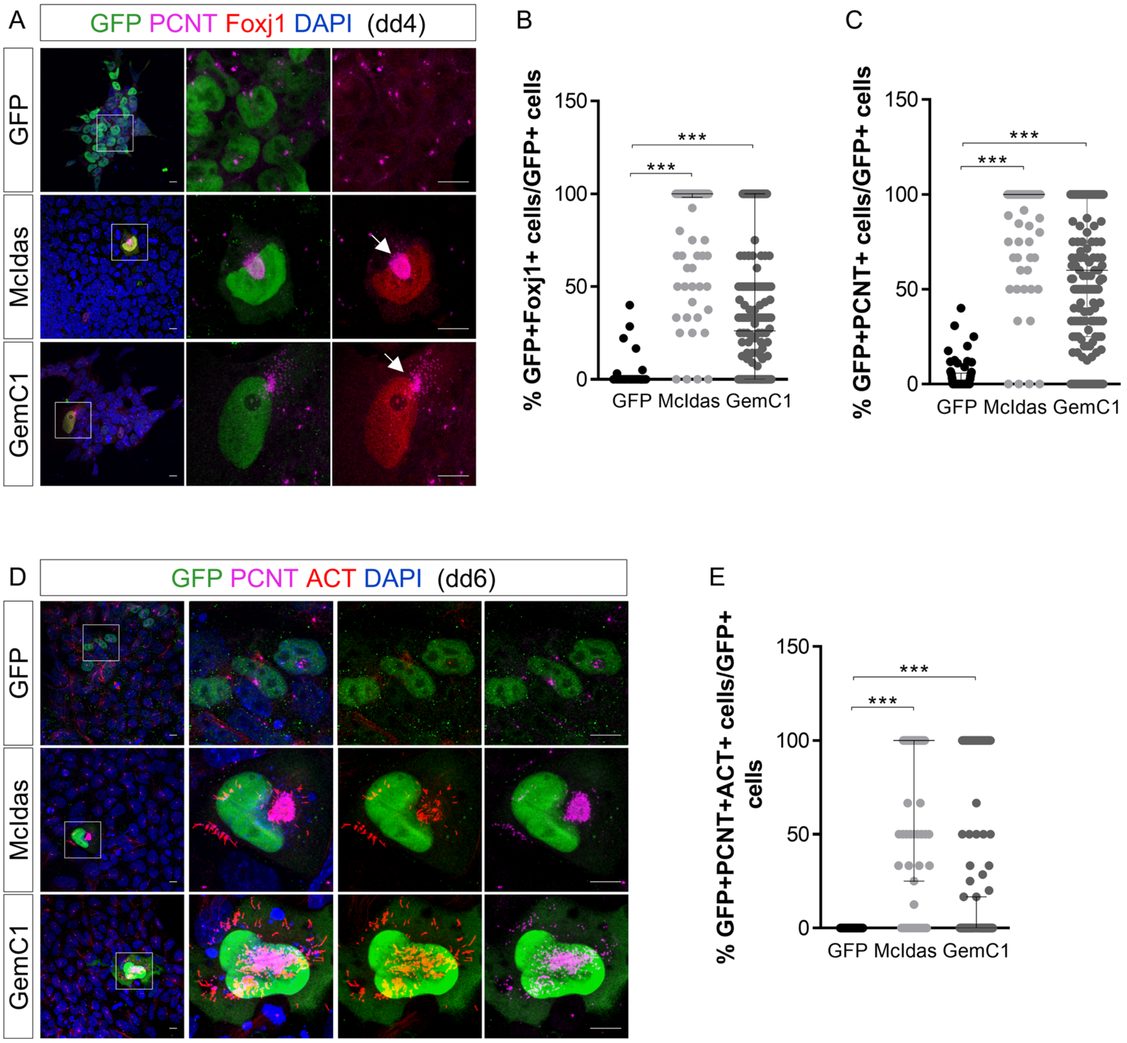

Figure EV1. Mouse embryonic stem cells programming into ependymal cells.

(**A**) Mouse embryonic stem cells (mESCs) were transfected with GFP, GFP-Mcldas (Mcldas) or GFP-GemC1 (GemC1) expressing vectors and subsequently cultured under differentiating conditions. Immunofluorescence was performed with antibodies against GFP (green), FoxJ1 (red) and Pericentrin (PCNT, magenta) at differentiation day 4. The arrow points to multiple basal bodies in Mcldas and GemC1 transfected cells which co-express the ependymal marker FoxJ1. Higher magnification of the boxed regions is shown in the right panels. (**B, C**) The graphs present the percentage of transfected mESCs that express FoxJ1 over the total number of the transfected cells (**B**) and the percentage of transfected cells which display multiple basal bodies based on the accumulation of PCNT signal. (**C**) Data are presented as the median ±IQR of two independent experiments. Statistical significance was determined using the nonparametric two-tailed Mann–Whitney test (***$P < 0.0001$). (**D**) GFP (green), PCNT (magenta) and acetylated α-tubulin (ACT red) immunostaining in transfected mESCs at differentiation day 6. Mcldas and GemC1 transfected mESCs present multiple basal bodies and cilia. (**E**) The graph shows the percentage of transfected cells with multiple basal bodies (PCNT accumulation) and multiple cilia (ACT labeled cilia) over the total number of the transfected cells. Data are presented as the median ±IQR of two independent experiments. Statistical significance was determined using the nonparametric two-tailed Mann–Whitney test (***$P < 0.0001$). DNA was stained with DAPI (blue). Scale bars, 10 µm. dd differentiation day.

© The Author(s)

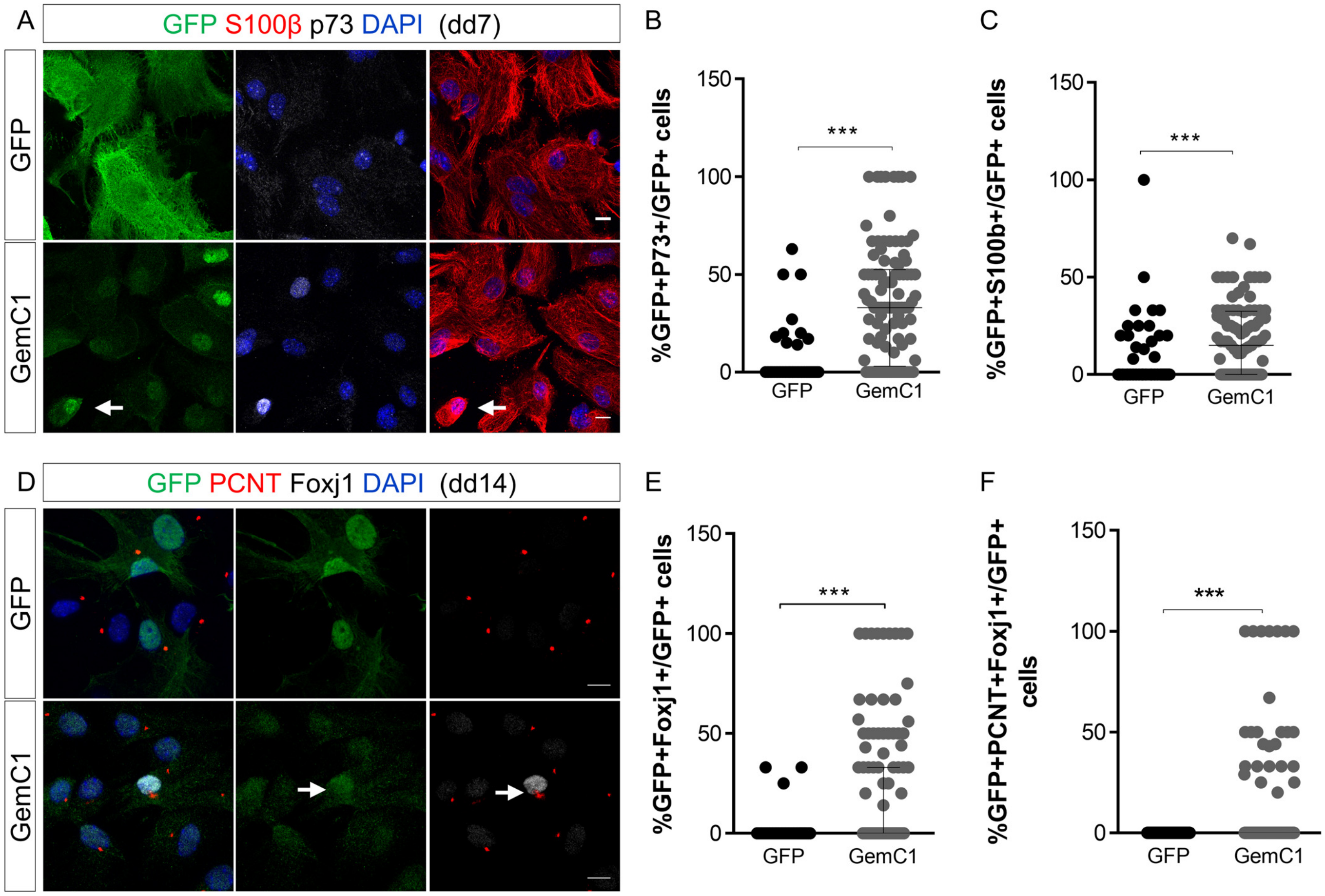

Figure EV2. GemC1 drives the commitment of murine cortical astrocytes towards the ependymal cell lineage.

(**A**) Cortical astrocytes were transduced with lentiviruses encoding GFP or GFP-GemC1 (GemC1). Immunofluorescence experiments were performed at day 7 of differentiation with specific antibodies against GFP (green) to mark the infected cells, P73 (gray) and S100β (red). Arrow point to a P73+ infected cell, committed to the ependymal lineage. (**B**, **C**) Graph depicting the percentage of infected cells which express P73 over the total number of infected cells (**B**). The graph shows the percentage of the infected cells which display S100β staining around their cell body (**C**). Three independent experiments were analyzed. Data are presented as the median ±IQR. Statistical significance was determined using the nonparametric two-tailed Mann–Whitney test (***$P < 0.0001$). (**D**) Astrocytes were immunostained against GFP (green), FoxJ1 (gray) and pericentrin (PCNT red) at differentiation day 14. The arrow points to a GemC1-infected cell which expresses FoxJ1 and possesses multiple basal bodies. (**E**, **F**) Graph presenting the percentage of the infected cells that express FoxJ1 over the total number of the infected cells. (**E**) The graph shows the percentage of infected cells which express the ependymal marker FoxJ1 and display accumulation of PCNT signal (**F**). Data are presented as the median ±IQR from three independent experiments. Statistical significance was determined using the nonparametric two-tailed Mann–Whitney test (***$P < 0.0001$). DNA was stained with DAPI (blue). Scale bars, 10 μm. dd differentiation day.

 © The Author(s)

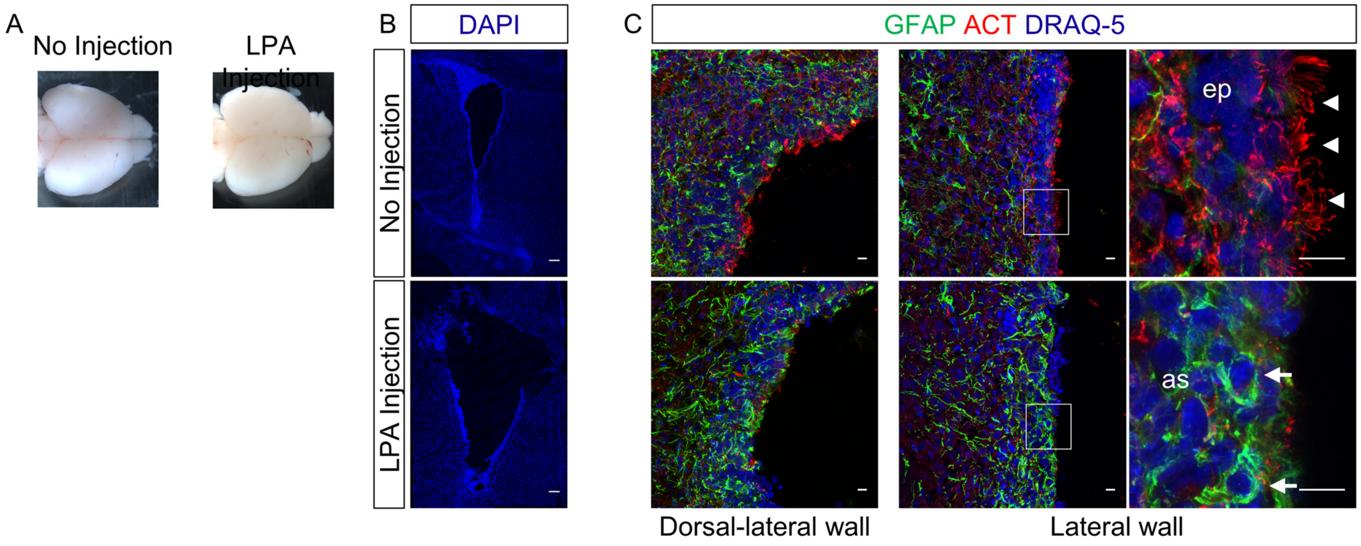

Figure EV3. Lysophosphatidic Acid (LPA) administration causes hydrocephalus in early postnatal stages in mice.

(**A**) Top views of Postnatal day 7 (P7) wild-type mouse brains that either received or not injection of LPA at the lateral ventricle at P5. No differences on the size of the brain were observed macroscopically. (**B**) DAPI staining on coronal brain sections from P7 mouse brains reveals the dilation of the lateral ventricles after LPA injections. Scale bar, 100 μm. (**C**) Immunofluorescence for Glial Fibrillary Acidic Protein (GFAP, green) which labels astrocytes and acetylated α-tubulin (ACT, red), a marker of ciliary axonemes on coronal sections from P7 mouse brains at the dorsal-lateral and lateral regions of the lateral walls. Arrowheads point to multiple cilia in control ependymal cells (ep). Arrows show GFAP-positive astrocytes (as) in the lateral wall of LPA-injected brains, where ciliary disruption is observed. Higher magnification of the boxed regions is shown in the right panels. Scale bars, 10 μm. DNA was stained with DAPI or Draq-5 (blue).

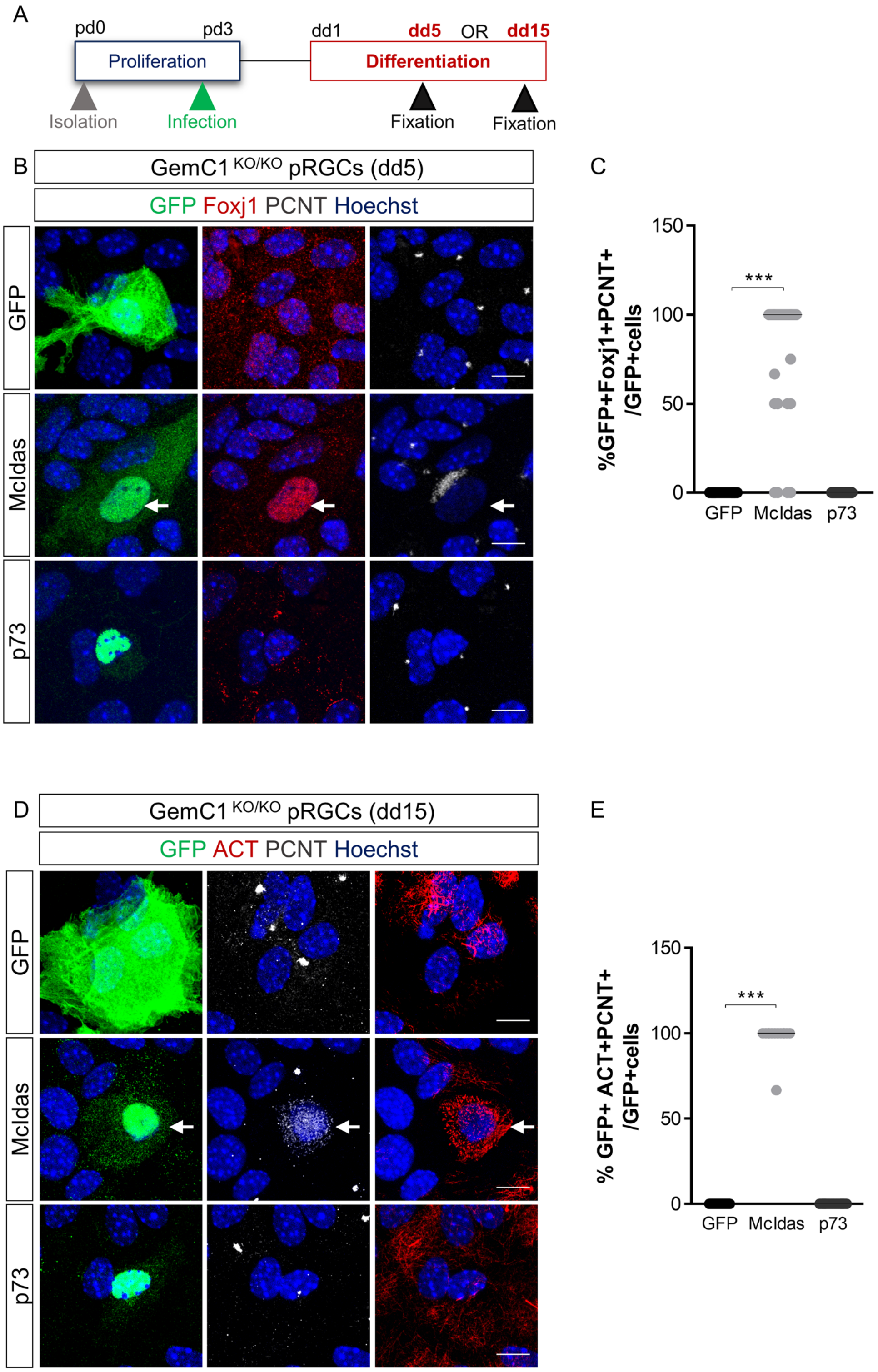
A
pd0
pd3
dd1
dd5
OR
dd15
Proliferation
Differentiation
Isolation
Infection
Fixation
Fixation
B
GemC1 KO/KO pRGCs (dd5)
GFP Foxj1 PCNT Hoechst
GFP
Mcldas
p73
C
%GFP+Foxj1+PCNT+ /GFP+cells
150
100
50
0
***
GFP Mcldas p73
D
GemC1 KO/KO pRGCs (dd15)
GFP ACT PCNT Hoechst
GFP
Mcldas
p73
E
% GFP+ ACT+PCNT+ /GFP+cells
150
100
50
0
***
GFP Mcldas p73

 © The Author(s)

Figure EV4. Ex vivo reprogramming of radial glial cells to ependyma can be achieved through McIdas ectopic expression.

(**A**) Schematic representation of experimental procedures for (B–E). Postnatal radial glial cells (pRGCs) were isolated from GemC1-knockout newborn mice, cultured and infected with lentiviruses expressing a GFP-McIdas (McIdas) or a GFP-P73 (P73) fusion protein, while GFP alone was used as a control. Cells were then cultured under differentiating conditions and analyzed at indicated time points. (**B**) Transduced radial glial cells were co-stained with antibodies against GFP (green) to mark infected cells, FoxJ1 (red) and pericentrin (PCNT, gray) 5 days after the initiation of differentiation. The arrow indicates the accumulation of PCNT signal in FoxJ1 expressing cells upon McIdas ectopic expression. (**C**) The graph presents the percentage of infected cells which express FoxJ1 and display multiple basal bodies (accumulation of PCNT signal). Three independent experiments were analyzed. Data are presented as the median ±IQR. Statistical significance was determined using the nonparametric two-tailed Mann–Whitney test (****P* < 0.0001). (**D**) Radial glial cells infected with GFP, McIdas, or P73 lentiviruses were labeled with antibodies against GFP (green), pericentrin (PCNT, gray) and acetylated α-tubulin (ACT, red) to detect mature multiciliated cells at differentiation day 15 (arrow). (**E**) The percentage of the infected cells which displayed multiple basal bodies, based on PCNT staining and simultaneously multiple cilia, based on ACT staining, was analyzed. Data are presented as the median ±IQR of three independent experiments. Statistical significance was determined using the nonparametric two-tailed Mann–Whitney test (****P* < 0.0001). DNA was stained with Hoechst (blue). Scale bars, 10 µm.

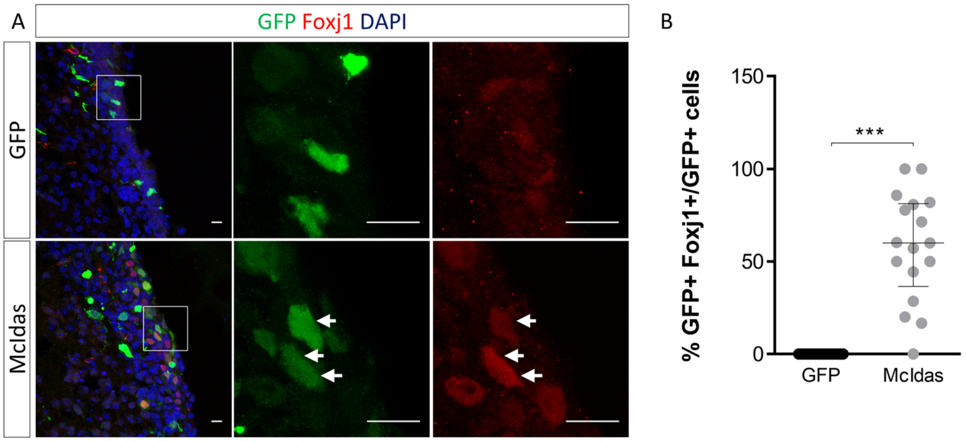

Figure EV5. Mcldas drives the commitment to the ependymal cell lineage in a genetic mouse model for hydrocephalus.

(**A**) Subventricular zone electroporation was conducted at postnatal day 1 (P1) GemC1-knockout mice with plasmids encoding GFP, or GFP-Mcldas (Mcldas). Coronal brain sections were stained with antibodies against GFP (green) to mark the electroporated cells and FoxJ1 (red), a marker of committed ependymal cells, 4 days post electroporation. Arrows point to Mcldas-electroporated cells expressing FoxJ1. Higher magnification of the boxed region is shown in the right panel. (**B**) Graph depicting the percentage of electroporated cells that express FoxJ1 over the total number of the electroporated cells. Two independent experiments were analyzed. Data are presented as the median ±IQR. Statistical significance was determined using the nonparametric two-tailed Mann–Whitney test (***$P < 0.0001$). DNA was stained with DAPI (blue). Scale bars, 10 µm.

 © The Author(s)
